# The moderating role of work environment in the relationship between proactive personality and personal growth initiative among nurses: a cross-sectional study

**DOI:** 10.1186/s12912-024-02040-6

**Published:** 2024-05-31

**Authors:** Xia Xie, Jie Tan, Jie He, Jiao Du, Chuan He, Zihao Li, Youchi Liu, Yuqin Chen

**Affiliations:** 1 Department of Nursing, Affiliated Hospital of North Sichuan Medical College, Nanchong, China; 2 School of Nursing, North Sichuan Medical College, Nanchong, China; 3 Department of Hepatic-biliary-pancreatic Surgery, Deyang People’s Hospital, Deyang, China; 4 Emergency Department, Affiliated Hospital of North Sichuan Medical College, Nanchong, China

**Keywords:** Personal growth initiative, Proactive personality, Hospital work environment, Nurses

## Abstract

**Backgrounds:**

Personal growth initiative (PGI) is regarded as a meaningful concept with potential value at both the individual and organizational levels, but little is known about the factors that contribute to nurses’ PGI. This study aimed to explore how proactive personality and hospital work environment affect PGI of clinical nurses.

**Methods:**

A cross-sectional study was conducted between September and October 2022 among 4414 nurses from 10 tertiary general hospitals in 10 cities in Sichuan, China, using a two-stage sampling method. Self-reported anonymous online questionnaires, such as sociodemographic information survey, personal growth initiative scale II, the 10-item proactive personality scale, and practice environment scale-nursing work index were used to collect data. Multiple hierarchical regression analyses were performed to examine research hypotheses.

**Results:**

Among the control variables in this study, nurses’ self-perceptions of general health status and professional title positively predicted PGI (*β* = 0.462, 95%CI = 0.272–0.653; *β* = 1.078, 95%CI = 0.508–1.648). After adding control variables, both proactive personality (*β* = 1.143, 95%CI = 1.096–1.190) and work environment (*β* = 3.391, 95%CI = 2.904–3.879) positively predicted PGI. The work environment positively moderated the association between proactive personality and PGI (*β* = 0.108, 95%CI = 0.025–0.191). These predictors jointly explained 50.3% of the variance in PGI.

**Conclusions:**

Nurses with a greater tendency to have a typical proactive personality have higher levels of personal growth initiative, and this positive effect will be better highlighted in a healthier work environment. Nursing managers should prioritize the employment of people with proactive personality traits, focus on the development and stimulation of proactive personality traits in nurses, and establish a supportive work environment to maximize the personal growth initiative of nurses.

## Backgrounds

Today, health care requirements and standards are increasing around the world. Nurses, as an important group in healthcare system, must keep up with the rapid advances in technology and knowledge to provide better patient care and to promote individual career development. Therefore, efforts to maintain personal growth initiative (PGI) in nursing profession are highly essential, as it is the main driving force of personal and professional development. PGI refers to the process of actively and voluntarily participating in personal growth [1, 2]. Robitschek et al. [1] confirmed the four components of PGI, including readiness for change, planfulness, resource utilization, and intentional behavior. readiness for change means being willing to undergo self-improvement. Planfulness emphasizes the understanding and practice of the planning process for self-improvement. The use of resources indicates that the individual use resources outside of oneself to assist in self-improvement, and intentional behavior refers to purposeful behavior in order to improve themselves. With the popularity of positive psychology, PGI, the positive and proactive attitude and behavior of individuals towards continuous self-improvement, play a crucial role in both individual careers and organizational human resources management. It is considered to be a prerequisite for the continuous growth of individuals in the organization, and is of great importance to both individuals and organizations [3]. On the one hand, PGI has a persistent positive effect on the individual’s optimal functioning and development [4]. Previous research has shown that individuals with a high level of PGI are more likely to find and take advantage of opportunities for self-growth than those with a low PGI level [5]. They also have better interpersonal relationships, a better sense of autonomy, stronger adaptive coping skills to overcome challenges and stress [1, 6], and lower levels of social anxiety and fear [7]. They are more likely to attain career success [8]. On the other hand, organizations also benefit from high levels of PGI of employees, because employees who are positive about personal growth are more able to stay healthy, energetic and productive in the face of demanding environments. Previous studies have shown that people with higher levels of PGI have better organizational identification [9], lower turnover intentions [10], higher levels of work engagement [10, 11], and better performance for the organization [8].

Although PGI is recognized as a valuable construct of potential importance at both individual and organizational levels, there is still little literature regarding factors responsible for PGI. The predictors of PGI have mostly been explored in terms of personal factors, including gender, age, education level, years of working, and personality [12–14], while only limited study has examined the effects of organizational variables. For example, Joo and colleagues [15] found that work empowerment, individual-organization fit and authentic leadership were positive predictors of PGI. However, no research has evaluated the combined effects of personal traits and work environment on PGI or the moderating effect of work environment on the relationship between personal traits and PGI. Furthermore, the existing research on PGI is mainly concentrated in the fields of education [16], psychology [17] and company management [15]. There are no reports of PGI research on hospital nurses, so little is known about how to develop PGI in this population. Understanding the relationships between nurses’ personal traits, work environment and PGI is important for developing future interventions. This study aimed to explore how proactive personality and hospital work environment affect PGI of clinical nurses.

### Proactive personality and PGI

Previous empirical studies have demonstrated that personality is one of the key predictors of PGI [12]. Personality Type A was shown to be positively correlated with PGI, whereas Personality Type B was found to be negatively associated with PGI [12]. However, the relationship between proactive personality, which is regarded as a unique personality trait not addressed in other personality theories, and PGI is still uncertain. According to Bateman and Crant [18], proactive personality is a trait that is characterized by an individual’s tendency to take proactive behaviors to influence their surroundings. Proactive personality may seem to be conceptually similar to PGI; however, it is actually different from PGI in that it refers to personal tendencies to influence environmental change [18, 19], whereas PGI focuses on intentional self-improvement cognition and behaviors [1]. Despite the fact that little research has directly examined the association between proactive personality and PGI, many scholars have explored and confirmed that proactive personality is positively correlated with proactive behavior and career success [20, 21]. Therefore, proactive personality can be assumed to be a positive predictor of PGI.

### Work environment, proactive personality and PGI

Although the effect of work environment on PGI has not been explored in the context of nursing, empirical research in the field of company management shows that workers have a higher level of PGI when they believe themselves to be a good match for the organization and are empowered at work [15], it shows that a better working environment is beneficial to improve the PGI level of individuals. Trait activation theory points out that the influence of individual traits on individual behaviors will be moderated by related situational factors [22]. Furthermore, from the perspective of social constructivism, personal career development is a socially constructed process that reflects not only personal traits but also the interaction of personal attributes with their surroundings [23, 24]. Therefore, it can be speculated that the predictive effect of individual characteristics on PGI is likely to be affected by some boundary conditions, but to our knowledge, there is no empirical research to confirm this hypothesis.

**Fig. 1:**
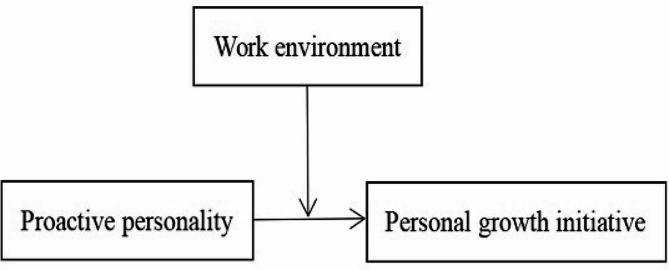
The conceptual framework diagram of this study

This study takes proactive personality as an individual trait that affects PGI. Nursing practice environment is the workplace characteristics that promote or hinder professional nursing practice, including nurse participation in hospital affairs, nursing foundations for quality of care, nurse manager ability, leadership and support of nurses, staffing and resource adequacy, and collegial nurse-physician relations, as situational factors or boundary conditions affecting the proactive personality and PGI relationship. Finally, a conceptual research framework is formed (Fig. 1) and the research hypotheses were as follows:

#### H1

Proactive personality positively predict PGI of clinical nurses.

#### H2

Nursing work environment plays a positive moderating role in the relationship between proactive personality and PGI.

## Methods

### Design, participants and procedures

This study used a cross-sectional design. Registered nurses from ten tertiary general hospitals in Sichuan Province, China were recruited to take part in the study. Based on the estimated total PGI score from the pre-survey (standard deviation = 9.44) and a 0.30 allowable level of error with 95% confidence intervals, the sample size for our study was computed by the NCSS-PASS 11.0, and a sample size of 3804 was needed. Taking into account a potential non-response rate of 20%, the final sample size was modified to 4565.

In our study, we used a two-stage sampling procedure. First, two tertiary general hospitals were selected randomly from each economic area of Sichuan Province (there are five economic regions: Chengdu Plain, Northeast Sichuan, Northwest Sichuan, South Sichuan and Panxi Economic Zone). Second, units within each of the selected hospitals were chosen at random. The ratio of clinical registered nurses among the chosen hospitals and the average number of clinical registered nurses in each clinical unit of the selected hospitals were used to determine the number of units to be sampled from each hospital (nurses’ manpower data were obtained through hospitals’ administration). All registered nurses in sampled units who satisfied the following criteria were enrolled: aged 18–60 years, with at least one year of nursing experience, and presently working in direct patient care roles. Nurses who were absent from duty during the survey, such as those on sick leave or maternity leave, were excluded.

The data were collected from eligible nurses using an online survey platform (Wenjuanxing). Each hospital had a research assistant (usually an assistant to the director of the nursing department) assigned to motivate participation in the study. All the research assistants received consistent training from the researcher on the purpose and significance of the research, the guiding language used in filling out the questionnaire, etc. Data collection was performed from September to October 2022. Each participant was required to complete the online questionnaire independently after signing an online written informed consent. If the data were incomplete or missing, participants were unable to submit their questionnaires through the survey platform. Also, only one submission per account or IP address was permitted. Consequently, 4414 of 4608 nurses (95.8%) completed this study successfully and voluntarily.

### Measures

#### PGI

PGI was measured using the personal growth initiative scale II (PGIS-II), which was developed by Robitschek et al. [1] and culturally modified by Li [13] among Chinese employees. The scale has three dimensions: readiness for change, using resources, and intentional behavior. There are 14 items in total, and each item is scored using a 6-point Likert scale [13]. The total score is the sum of the scores for all items, with a total score ranging from 0 to 70 [13]. Higher scores indicate higher levels of PGI. The scale has satisfactory structural validity and a Cronbach’s alpha of 0.820 to 0.870 for each domain [13]. In this study, the Cronbach’s alpha for each domain ranged from 0.83 to 0.93.

#### Proactive personality

The 10-item proactive personality scale, which was made by Seibert et al. [25] and adapted by Yan [26] for Chinese culture, was used to measure proactive personality. It is scored on a 5-point Likert scale from 1 (absolutely disagree) to 5 (absolutely agree), with a higher score indicating a greater tendency for an individual to have a typical proactive personality [27]. Previous studies have shown that the scale has good reliability and validity in Chinese nurses, with a Cronbach’s alpha of 0.831 [27]. The Cronbach’s alpha for the scale was 0.877 in this study.

#### Work environment

The practice environment scale-nursing work index (PES-NWI), which was developed by Lake [28] and culturally modified by Zheng [29], was used to assess work environment. The scale has five subscales with a total of 29 items. Each item is scored on a 4-point Likert scale from 1 (strongly disagree) to 4 (strongly agree), with higher scores showing a better perceived work environment [29]. Previous studies have shown that the scale has acceptable reliability and validity in Chinese nurses, with a Cronbach’s alpha of 0.74 to 0.86 for each subscale [29]. The Cronbach’s alpha for each subscale ranged from 0.828 to 0.927 in this study.

#### Control variables

In this study, the demographic variables and individual information of nurses were investigated as control variables, including gender, age, marital status, highest degree, children information, general health status, years of nursing experience, work units, employment types, and professional title.

### Data analysis

Participants’ sociodemographic characteristics and variable scores were described by frequency (percentage) and mean (standard deviation), as appropriate. Differences in PGI total scores between subgroups were examined using *t* tests or one-way ANOVA. The associations between continuous variables were explored using Pearson’s correlation analysis. Hierarchical moderated regression analysis using PGI as dependent variables were used to assess the research hypotheses. Control variables were entered in the first step, followed by proactive personality and work environment in the second step. The interaction term “proactive personality x work environment” was then added to the final model to test the moderating effect of the work environment. As suggested by Aiken and West [30], predictors including proactive, work environment and interaction term (proactive personality x work environment) were centralized around the mean prior to hierarchical regression analysis in order to reduce multicollinearity. Simple slope diagrams based on the method proposed by Cohen and colleagues [31] were plotted to visualize the moderating effect of the work environment. All analyses were performed with SPSS version 27 and the SPSS add-on PROCESS v4.0. A *P*-value of less than 0.05 was considered statistically significant.

## Results

### Characteristics of sample

Tables 1 and 2 show sample demographic information as well as descriptive statistics on key variables. The majority of participants were female nurses, with an average age of 31.54 and 9.75 years of nursing experience. Over 2/3 of the participants were junior nurses (76.7%) and obtained a bachelor’s degree or higher (78.3%). Slightly over 1/4 of the samples were in fair or poor health. The average score of PGI, proactive personality and work environment was 49.02, 37.99 and 3.15, respectively.

**Table 1:** Demographic information of participants and differences in PGI scores

	*N* (%)	PGI (M ± SD)	F/t
**Gender**			1.787
Female	4266 (96.6%)	49.06 ± 9.28	
Male	148 (3.4%)	47.68 ± 9.64	
**Marital status**			-3.286**
Single	1042 (23.6%)	48.19 ± 9.51	
Married	3372 (76.4%)	49.27 ± 9.22	
**Highest degree**			-2.034*
Junior college or below	956 (21.7%)	48.48 ± 9.46	
Bachelor or above	3458 (78.3%)	49.17 ± 9.25	
**General health**			-41.393***
Poor	52 (1.2%)	44.00 ± 11.85	
Fair	1098 (24.9%)	46.83 ± 9.01	
Good	1168 (26.4%)	48.76 ± 8.74	
Very good	1408 (31.9%)	49.60 ± 9.37	
Excellent	688 (15.6%)	52.13 ± 9.25	
**Children information**			9.159***
Childless	1384 (31.3%)	48.25 ± 9.25	
Infancy	1300 (29.5%)	49.07 ± 9.34	
Preschool stage	890 (20.2%)	48.74 ± 9.50	
School stage	408 (9.2%)	49.75 ± 9.14	
Other	432 (9.8%)	51.19 ± 8.67	
**Units**			5.688***
Medical units	1848 (41.9%)	49.10 ± 9.46	
Surgical units	1770 (40.1%)	49.47 ± 9.32	
Emergency units	242 (5.5%)	46.69 ± 8.84	
Intensive care units	230 (5.2%)	48.09 ± 8.40	
Other units	324 (7.3%)	48.53 ± 8.88	
**Employment types**			
Permanent	848 (19.2%)	50.23 ± 9.43	4.227***
Temporary	3566 (80.8%)	48.73 ± 9.24	
**Professional title**			24.212***
Junior title	3386 (76.7%)	48.54 ± 9.33	
Intermediate title	872 (19.8%)	50.21 ± 9.17	
Senior title	156 (3.5%)	52.72 ± 7.69	

Note: *P < 0.05; **P < 0.01;***P < 0.001; PGI: Personal growth initiative; M ± SD: mean ± standard deviation

**Table 2:** Statistical description of continuous variables and correlation between variables

Variables	M	SD	1	2	3	4	5
1. Age	31.54	6.10	-				
2. Years of nursing experience	9.75	7.08	0.954***	-			
3. Personal growth initiative	49.02	9.30	0.059***	0.060***	***(0.954)***		
4. Proactive personality	37.99	4.85	0.152***	0.150***	0.687***	***(0.877)***	
5. Work environment	3.15	0.45	0.056***	0.052***	0.453***	0.463***	***(0.970)***

Note: *P < 0.05; **P < 0.01;***P < 0.001; PGI: Personal growth initiative; M ± SD: mean ± standard deviation

Reliability coefficients appear in bold italic along the diagonal

### Associations between study variables

Nurses with different demographic information, except for gender, had statistically significant differences in total PGI scores (Table 1). Results of the Pearson correlation between continuous variables were shown in Table 2. Age, years of nursing experience, proactive personality, and work environment were positively associated with PGI.

### Results of regression analysis

As shown in Table 3, among the control variables in this study, nurses’ self-perceptions of general health status and professional title positively predicted PGI (*β* = 0.462, 95%CI = 0.272–0.653; *β* = 1.078, 95%CI = 0.508–1.648). After adding the control variables, proactive personality and work environment was positively correlated with PGI (*β* = 1.143, 95%CI = 1.096–1.190; *β* = 3.391, 95%CI = 2.904–3.879). The interaction of proactive personality and work environment had a significant positive correlation with PGI (*β* = 0.108, 95%CI = 0.025–0.191), that is, work environment has a positive moderating effect on the correlation between proactive personality and PGI. The above-mentioned predictors jointly explained 50.3% of the variance in PGI (Table 3).

**Table 3:** Work environment as the moderating role: proactive personality as the independent variable in regression analysis

Variables	Model 1			Model 2			Model 3	
	β	95% CI		β	95% CI		β	95% CI
Gender	-0.551	[-2.113 to 1.010]		-0.523	[-1.622 to 0.577]		-0.539	[-1.638 to 0.560]
Age	0.068	[-0.149 to 0.286]		0.133	[-0.023 to 0.290]		0.136	[-0.021 to 0.292]
Years of nursing experience	0.065	[-0.065 to 0.196]		0.036	[-0.059 to 0.130]		0.036	[-0.059 to 0.130]
Marital status	0.483	[-0.257 to 1.222]		0.356	[-0.180 to 0.891]		0.335	[-0.201 to 0.870]
Highest degree	0.54	[-0.129 to 1.210]		0.592	[-0.096 to 1.279]		0.605	[-0.082 to 1.292]
General health	1.689***	[1.432–1.947]		0.460***	[0.269–0.651]		0.462***	[0.272–0.653]
Children information	0.411*	[0.047–0.775]		0.072	[-0.192 to 0.336]		0.085	[-0.179 to 0.349]
Units	-0.134	[-0.368 to 0.099]		-0.018	[-0.187 to 0.151]		-0.023	[-0.192 to 0.146]
Employment types	-0.772	[-1.605 to 0.062]		-0.424	[-1.029 to 0.181]		-0.436	[-1.041 to 0.169]
Professional title	2.028***	[1.242–2.814]		1.085***	[0.514–1.655]		1.078***	[0.508–1.648]
Proactive personality				1.155***	[1.109–1.201]		1.143***	[1.096–1.190]
Work environment				3.441***	[2.954–3.927]		3.391***	[2.904–3.879]
Proactive personality × Work environment							0.108*	[0.025–0.191]
R^2^	0.052			0.502			0.503	
∆R^2^	0.052***			0.450***			0.001*	

Note: *P < 0.05; **P < 0.01;***P < 0.001; 95%CI: 95% confidence interval

**Fig. 2:**
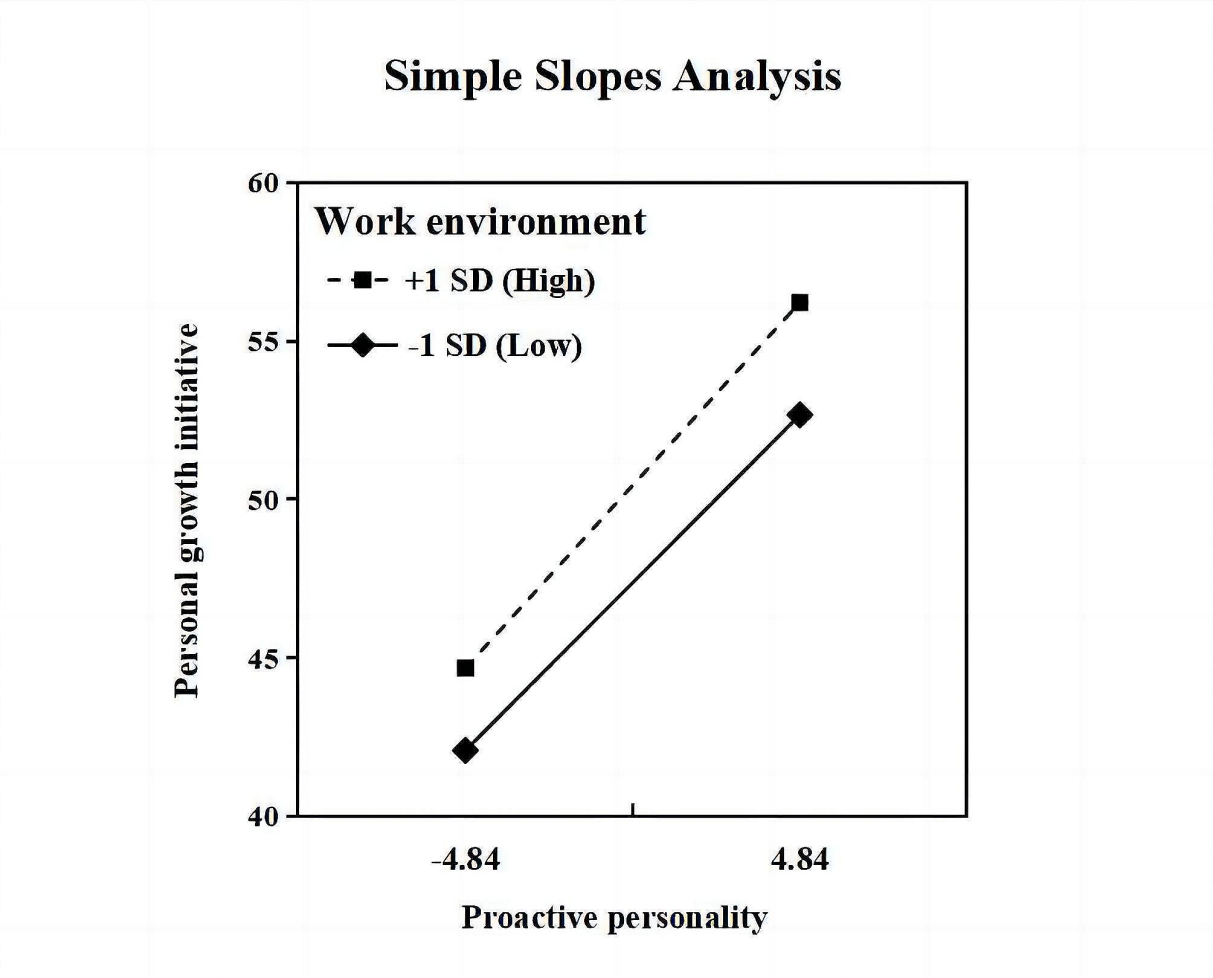
The moderating effect of work environment on the relationship between proactive personality and PGI.

When the moderating effect was depicted, it was also found that the effect of proactive personality on PGI increases as work environment become healthier (Fig. 2). There was an increasing trend in coefficients in work environment one standard deviation below the mean (*β* = 1.09, 95%CI = 1.03–1.16), at the mean (*β* = 1.14, 95%CI = 1.10–1.19), and one standard deviation above the mean (*β* = 1.19, 95%CI = 1.14–1.25).

## Discussion

This study aimed to explore the relationships among proactive personality, work environment and PGI. We believe that it would contribute to the existing literature in several ways. First, this study used data from Chinese hospital nurses to introduce PGI, which was a relatively less explored concept, particularly in the context of nursing work. Second, this study explored the determinants of PGI in terms of personal traits and work environments. It should be noted that no studies to date have examined their integrative effects on PGI. Lastly, this study attempted to unveil the process of how the determinants interact with each other through a moderated research model. The findings support the study hypotheses that proactive personality positively predicts PGI after adding control variables, and that the work environment, as a potential moderator, plays an important role in the relationship between proactive personality and PGI. These results will be discussed in the following paragraphs.

The current finding confirms that proactive personality positively predicts PGI. Specifically, nurses with a greater tendency to have a typical proactive personality have a higher level of PGI. The result supports the findings of similarity studies conducted in the field of companies, which indicated that employees with proactive personality are more likely to engage in active behaviors at work [20]. These findings can be explained by action regulation theory, which believes that proactive personality can influence individual behaviors by improving prospects of action regulation at work [11]. According to the theory, proactive personality may be a strong predictor of nurses’ PGI, because it creates favorable prospects for nurses to prepare for and implement self-improvement plans. Considering the important role of PGI in individual nurses and nursing in general, it is recommended that when recruiting and training nurses, managers may give priority to hiring individuals with proactive personality traits, or place emphasis on the cultivation and stimulation of their proactive personality traits in the process of training.

Analogously with other research [15], our study found that work environment was positively correlated with PGI. Previous studies have found that a healthy workplace has chances for nurse engagement and participation in hospital administration, a nursing model of care delivery, supportive nurse leaders, great nurse-physician interactions, and enough nurse staffing and resources, which are also considered to be common working environment characteristics of magnet hospitals [28, 32]. Furthermore, Lake and colleagues [33] analyzed the relationships among nursing work environment and job and health outcomes, and found that work environment was related with lower odds of negative nurse and patient outcomes, and poor safety or quality ratings, but higher odds of patient satisfaction. The contribution of this study is that it is the first known study to reveal the combined effects of proactive personality and work environment on the prediction of PGI. The result showed that work environment positively moderated the relationship between proactive personality and PGI. This finding seems to suggest that a healthier work environment is more conducive to the strengths of nurses with proactive personality, in other words, positive effect of proactive personality on PGI will be better highlighted when nurses are in a healthier work environment. At the same time, a better work environment may inspire those nurses with non-proactive personality, which is conducive to improving the level of PGI. These findings support trait activation theory and the career development theory of constructivism, which views individuals’ behaviors as a result of the interaction between personal traits and work environments (Tett et al., 2000; Bandura, 1986; Chen, 2003). This study reemphasizes the importance of establishing a healthy work environment from the new perspective of enhancing the positive predictive effect of proactive personality on PGI.

Last but not least, this study found that self-perceived general health status and professional title were positively associated with nurses’ PGI. This indicates that nurses with better self-perceived general health and higher rank of professional title have higher levels of PGI. The reasonable explanation was that individual’s behavior is triggered by demands [34]. Self-improvement at work belongs to the category of self-actualization needs, while health is a basic human need. According to Maslow’s theory of hierarchy of needs, individuals develop higher needs only after the lower ones are satisfied [34]. Previous research has shown that those with high levels of PGI have a greater likelihood of career progression [8]. According to social interaction theory, if individuals have the experience of being rewarded for undertaking certain behaviors, they will positively reinforce those behaviors [35]. Therefore, it can be speculated that individuals with a high level of PGI are more likely to obtain higher rank of professional title, and then strengthen their PGI from this beneficial experience. These findings suggest that managers should also consider personal factors, such as professional titles and health status, in the process of developing nurses’ PGI.

### Limitations and future research

There are several possible limitations to the study. First, this study used a cross-sectional design, which cannot identify possible developmental changes in proactive personality, perceived work environment and PGI. Future studies should focus on longitudinal investigations. Second, most of the participants were female nurses from tertiary general hospitals in a specific province (Sichuan) in China, which may limit the generalizability of the findings. The applicability of our findings to nursing workplaces that include more junior nurses and more male nurses, as well as to other categories of hospital nurses, still needs further evaluation in future studies.

### Relevance to clinical practice

The exploration of the relationships among the three variables of proactive personality, work environment and PGI has provided new insights for health care administrators and nursing managers to develop hospital nurses’ PGI. These findings have implications for nurses’ PGI development interventions. Strategies such as prioritizing the recruitment of people with proactive personality traits, emphasizing the development and stimulation of proactive personality traits in nurses, establishing a supportive working environment, caring for health status of nurses, and helping them promote professional titles may be effective methods for improving nurses’ PGI.

## Conclusions

Promoting the development of PGI for nurses is of great importance both for their own career development and for organizational success. Our study in a large population of Chinese nurses confirms that nurses with greater tendency to be typical proactive personality have higher levels of PGI; and when nurses in a healthier work environment, positive effect of proactive personality on PGI will be better highlighted. These findings provide a new insight for health care administrators and nursing managers to develop strategies to develop hospital nurses’ PGI.

## Data Availability

The datasets used and/or analysed during the current study are available from the corresponding author on reasonable request.

## Abbreviations

PGI: Personal growth initiative
PGIS-II: Personal growth initiative scale II
PES-NWI: Practice environment scale-nursing work index
